# Phylogenomic and Comparative Analyses of Complete Plastomes of *Croomia* and *Stemona* (Stemonaceae)

**DOI:** 10.3390/ijms19082383

**Published:** 2018-08-13

**Authors:** Qixiang Lu, Wenqing Ye, Ruisen Lu, Wuqin Xu, Yingxiong Qiu

**Affiliations:** Key Laboratory of Conservation Biology for Endangered Wildlife of the Ministry of Education, and College of Life Sciences, Zhejiang University, Hangzhou 310058, China; 0016616@zju.edu.cn (Q.L.); yewenqing@zju.edu.cn (W.Y.); reason@zju.edu.cn (R.L.); 21707105@zju.edu.cn (W.X.)

**Keywords:** *Croomia*, *Stemona*, chloroplast genome, comparative genomics, phylogeny, biogeography

## Abstract

The monocot genus *Croomia* (Stemonaceae) comprises three herbaceous perennial species that exhibit EA (Eastern Asian)–ENA (Eastern North American) disjunct distribution. However, due to the lack of effective genomic resources, its evolutionary history is still weakly resolved. In the present study, we conducted comparative analysis of the complete chloroplast (cp) genomes of three *Croomia* species and two *Stemona* species. These five cp genomes proved highly similar in overall size (154,407–155,261 bp), structure, gene order and content. All five cp genomes contained the same 114 unique genes consisting of 80 protein-coding genes, 30 tRNA genes and 4 rRNA genes. Gene content, gene order, AT content and IR/SC boundary structures were almost the same among the five Stemonaceae cp genomes, except that the *Stemona* cp genome was found to contain an inversion in *cem*A and *pet*A. The lengths of five genomes varied due to contraction/expansion of the IR/SC borders. A/T mononucleotides were the richest Simple Sequence Repeats (SSRs). A total of 46, 48, 47, 61 and 60 repeats were identified in *C. japonica*, *C. heterosepala*, *C. pauciflora*, *S. japonica* and *S. mairei*, respectively. A comparison of pairwise sequence divergence values across all introns and intergenic spacers revealed that the *ndh*F–*rpl*32, *psb*M–*trn*D and *trn*S–*trn*G regions are the fastest-evolving regions. These regions are therefore likely to be the best choices for molecular evolutionary and systematic studies at low taxonomic levels in Stemonaceae. Phylogenetic analyses of the complete cp genomes and 78 protein-coding genes strongly supported the monophyly of *Croomia*. Two Asian species were identified as sisters that likely diverged in the Early Pleistocene (1.62 Mya, 95% HPD: 1.125–2.251 Mya), whereas the divergence of *C. pauciflora* dated back to the Late Miocene (4.77 Mya, 95% HPD: 3.626–6.162 Mya). The availability of these cp genomes will provide valuable genetic resources for further population genetics and phylogeographic studies on *Croomia.*

## 1. Introduction

*Croomia* Torr. ex Torr. et Gray belongs to the monocot family Stemonaceae Engl (Pandanales, Liliidae) and comprises three herbaceous perennial species: *C. pauciflora* (Nutt.) Torr., *C. japonica* Miq. and *C. heterosepala* (Bak.) Oku. Of these three species, *C. japonica* and *C. heterosepala* are endemic to warm-temperate deciduous forests in East Asia, while *C. pauciflora* grows in temperate-deciduous forests in North America [1,2,3]. There is a considerable difference in morphological traits among this genus. For example, the four tepals of *C. japonica* are homomorphic with a re-curved edge, while those of *C. heterosepala* have a flat edge, and one outside tepal is much larger than the other three [4,5]. ­Compared to two Asian species, *C. pauciflora* has a smaller flower, shorter petiole, denser underground stem nodes and a more obvious heart-shape leaf base [1]. As the roots of *Croomia* species contain compounds such as pachysamine, didehydrocroomine and croomine groups, they are used as folk medicine to treat cough and injuries [6,7]. *Croomia* can reproduce sexually through seed formation via cross-pollination and asexually through underground rhizomes [1,8]. Due to their limited distribution and small population sizes, the three extant species of *Croomia* are listed as “threatened” or “endangered” in China, Japan and the Americas [9,10,11]. The other three genera of Stemonaceae are *Pentastemona*, *Stemona* and *Stichoneuron*. The species of *Stichoneuron* are located in India, Thailand and Peninsular Malaysia, while those of *Pentastemona* are only in Sumatra [3,8]. The genus *Stemona* comprises ca. 25 species with the widest distribution from Northeast Asia to Southeast Asia and Australia. The roots of *Stemona* species contain similar medicine compounds as *Croomia* [7]. Although *Croomia* and *Stemona* species have important pharmacological and ecological value, limited molecular markers were available for the utilization, conservation and breeding of these species in the context of population genetics and phylogenetic studies [12].

*Croomia* exhibits a well-known classic intercontinental disjunct distribution between Eastern Asia (EA) and Eastern North America (ENA) [1,8,13,14]. This continental disjunction pattern was suggested to have resulted from fragmentation of the mid-Tertiary mesophytic forest flora throughout a large part of the Northern Hemisphere, as global temperature cooled down in the late Tertiary and Quaternary [15,16]. For the two East Asian endemics, *C. japonica* is distributed in E­ast China and southern Japan, while *C. heterosepala* is in northern Japan, and they have adjacent ranges in South Japan [17,18]. Therefore, *Croomia* is well suited for testing biogeographic hypotheses about the evolution of both the eastern Asian–eastern North American and eastern Asian–Japanese Archipelago floristic disjunctions. Based on previous molecular phylogenetic analyses using cpDNA sequence variation of the *trn*L-F region, the two Asian species were identified as sister species that likely diverged in the Mid-to-Late Pleistocene (0.84–0.13 million years ago, Mya), whereas the divergence of *C. pauciflora* dates back to the Late Plio-/Pleistocene (<2.6 Mya) [12]. However, the previous cpDNA analysis based on a few parsimony informative sites yielded low bootstrap values for the majority of clades [12]. Thus, it is necessary to develop more highly variable genetic markers for determining the phylogenetic relationships and divergence times for *Croomia*. Nowadays, many phylogenetic relationships that remained unresolved with few loci have been clarified by using whole cp genome sequences [19,20,21]. Thus, whole cp genome sequences are increasingly being used in phylogeny reconstruction and providing hypervariable genetic markers for population genetic studies, especially in a group of recently-diverged species [22,23]. 

Here, we sequenced three *Croomia* and two *Stemona* cp genomes using the next-generation Illumina genome analyzer platform. We compared the cp genomes of two Stemonaceae genera to characterize their structural organization and variations and identify the most variable regions. This information on interspecific variability of each region will help guide further systematic and evolutionary studies of Stemonaceae. In addition, we used the whole cp genomes to resolve the phylogenetic relationships of *Croomia* and infer the historical biogeography of the genus.

## 2. Results and Discussion

### 2.1. Genome Assembly and Features

**Figure 1:**
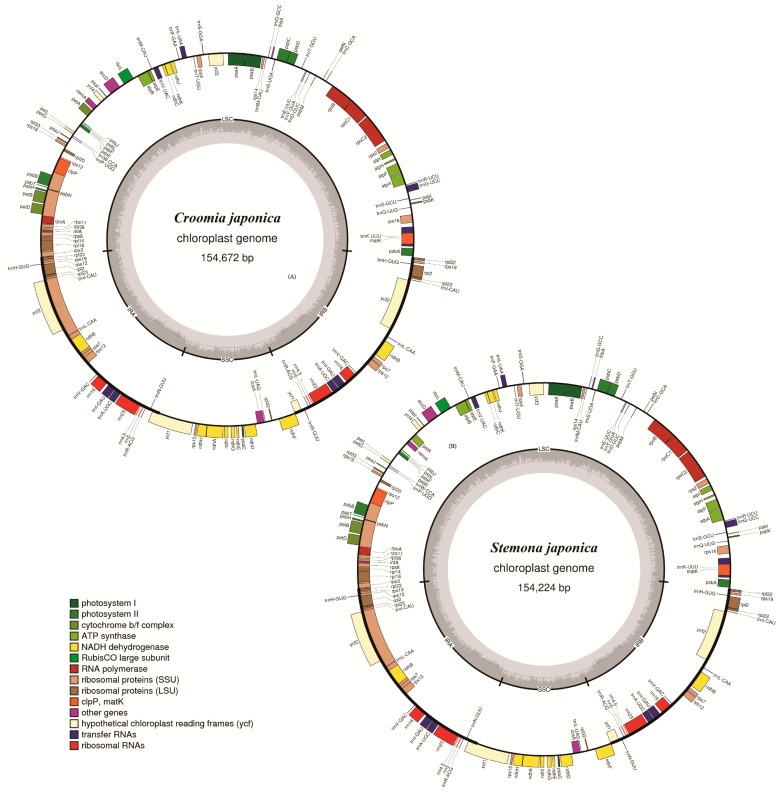
Gene maps of *Croomia* and *Stemona* chloroplast genomes. (**A**) *Croomia japonica*; (**B**) *Stemona japonica*.

Illumina paired-end sequencing yielded 14,163,520–31,094,272-bp clean reads after trimming, and the de novo assembly generated 50,369–123,479 contigs for five Stemonaceae species. With the cp genome of *C. palmata* as a reference, contigs were combined to generate the draft cp genome for each species. The lengths of determined nucleotide sequences were 154,672, 154,407, 155,261, 154,224 and 154,307 bp for *C. japonica*, *C. heterosepala*, *C. pauciflora*, *S. japonica* and *S. mairei*, respectively. (Figure 1, Table S1). All five cp genomes exhibited the typical quadripartite structure of angiosperms, consisting of a pair of IR regions (27,082–27,243 bp) separated by an LSC region (81,844–82,429 bp) and an SSC region (17,889–18,346 bp). The cp genomes of three *Croomia* species and two *Stemona* species were deposited in GenBank (MH177871, MH191379–MH191382).

These five cp genomes contained 134 genes identically, of which 114 were unique and 20 were duplicated in IR regions (Table S1). Those 134 genes were arranged almost in the same order except *cem*A and *pet*A, which were inverted at the LSC region of two *Stemona* species. Gene inversions at LSC were also reported in other angiosperm, such as *Silene* [24], *Cymbidium* [19] and *Acacia dealbata* [25]. The 114 unique genes included 80 protein-coding genes, 30 tRNA genes and 4 rRNA genes. In *Croomia* species, the overall GC content was 38.3%, and the GC contents of the LSC, SSC and IR regions were 36.6%, 32.3–32.5% and 42.8–42.9%, respectively, while those of *Stemona* were 38.0%, 36.2%, 32.1% and 42.7% (Table S1). In all five genomes, nine of the protein-coding genes and six of the tRNA genes possessed a single intron, while three genes (*rps*12, *clp*P and *ycf*3) contained two introns (Table 1). The *rps*12 gene was trans-spliced; the 5′ end exon was located in the LSC region, and the 3′ end exon and intron were located in the IR regions. Compared to many other species, such as *Salvia miltiorrhiza* [26] and *Cornales* [27], the SSC region of the five studied species was found to have a different (reverse) orientation. The reverse orientation of the SSC region has also been reported in a wide variety of plant species [28,29,30]. This phenomenon is sometimes interpreted as a major inversion existing within the species [29,31,32]. In fact, the two orientations of the SSC region have been found to occur regularly during the course of chloroplast DNA replication within individual plant cells [33,34]. Thus, the reverse orientation of the SSC region found in the five Stemonaceae cp genomes may represent a form of plastid heteroplasmy [30,35].

### 2.2. Contraction and Expansion of Inverted Repeats 

**Figure 2:**
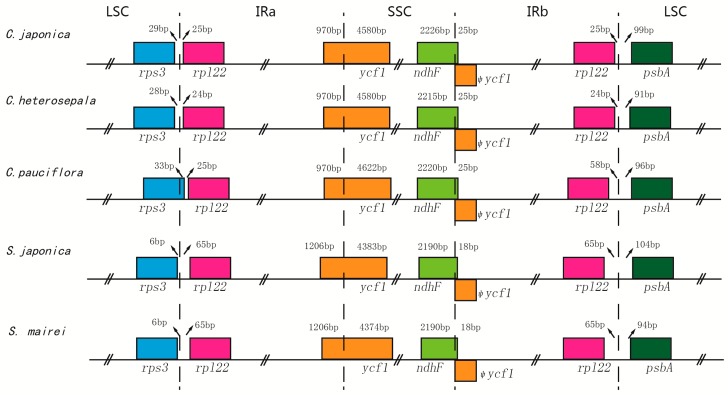
Comparison of LSC, IR and SSC junction positions among five Stemonaceae chloroplast genomes.

Length variation in angiosperm cp genomes is due most typically to the expansion or contraction of the IR into or out of adjacent single-copy regions and/or changes in sequence complexity due to insertions or deletions of novel sequences [36,37]. Compared to reference cp genome *C. palmata*, all five species exhibited IR expansion at the IRb/LSC border, leading to entire *rpl*22 duplication. In a previous study, a partial duplication of the *rpl*22 gene was reported in some monocot species of Asparagales and Commelinales [38]. Although the gene number and gene order were conserved across these five Stemonaceae species, minor differences were still observed at the boundaries (Figure 2). At the IRa/LSC border, the spacer from *rpl*22 to this border of *Stemona* (65 bp) was longer than that of *Croomia* (24–25 bp), except *C. pauciflora*. As for the *ycf*1 gene, there were 4580–4662-bp sequences located at SSC in *Croomia* and 4374–4383 bp in *Stemona*, while the pseudogene fragment duplications in IRb were 970 bp and 1206 bp in *Croomia* and *Stemona*, respectively. The *ndh*F gene exhibited variable sequences in SSC of *Croomia* (2215–2226 bp), while invariable in *Stemona* with a 2190-bp length. At the border of IRb/LSC, the spacer from *psb*A to this border of *Croomia* ranged from 91 bp–99 bp, while it ranged from 94 bp–104 bp in *Stemona*. These differences between the five cp genomes led to the length variation of their whole genome sequences.

### 2.3. Divergence Hotspot Regions

**Figure 3:**
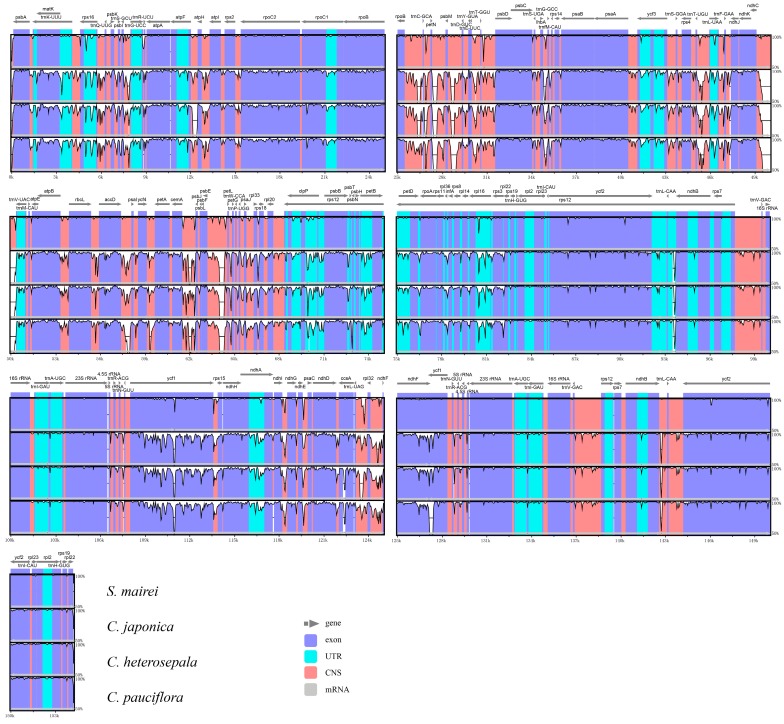
Sequence identity plots among five Stemonaceae chloroplast genomes, with *Stemona japonica* as a reference. CNS: conserved non-coding sequences; UTR: untranslated region.

**Figure 4:**
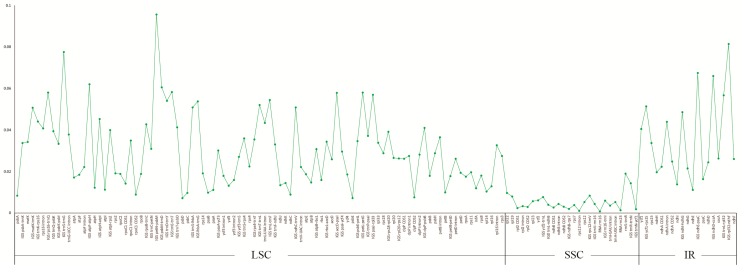
The nucleotide variability (Pi) values were compared among five Stemonaceae species.

**Figure A1:**
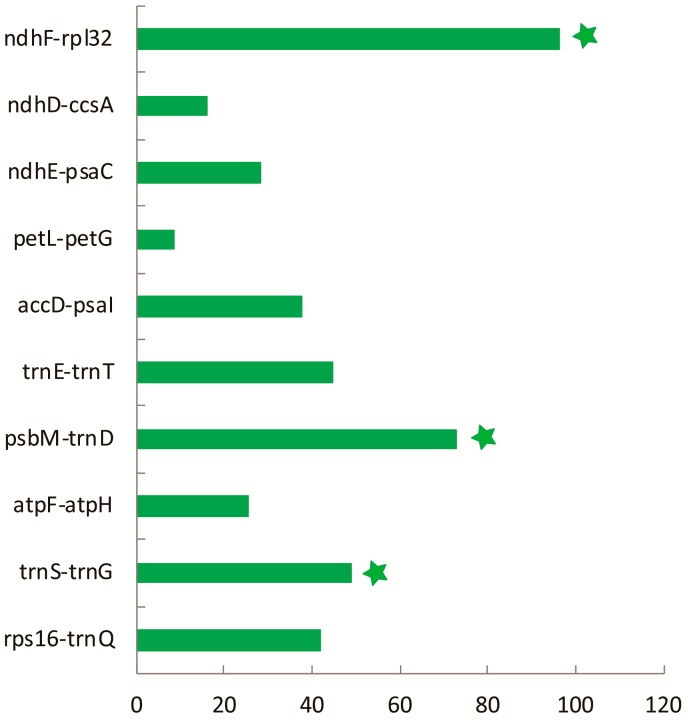
PIC values of the top ten most variable noncoding regions in Stemonaceae.

To elucidate the level of sequence divergence, the three *Croomia* and two *Stemona* cp genome sequences were compared and plotted using the mVISTA program (Figure 3). Like most angiosperms, the sequence divergence of IR regions was lower than that of the LSC and SSC region [39,40], which may involve copy correction of IRs as a mechanism [41]. We identified 140 regions in total with more than a 200-bp length (68 protein-coding regions (CDS), 53 Intergenic Spacers (IGS) and 19 introns). The nucleotide variability (Pi) of these 140 regions ranged from 0.080% (*rrn*16) to 9.565% (IGS *pet*N–*psb*M) among the five cp genomes. The average Pi of the non-coding region was 3.644%, much higher than coding regions (1.587%), as found in most angiosperms [42,43]. For the 68 CDS, the Pi values for each region ranged from 0.231% (*rpl2* CDS1) to 4.047% (*ycf*1), whereby 10 regions (i.e., *mat*K, *rpl*33, *rps*15, *psb*H, *rps*18, *rps*3, *rpl*20, *ccs*A, *ndh*F, *acc*D) had remarkably high values (*pi* > 2.5%). For the 53 IGS regions, Pi values ranged from 0.185% (*trn*N–*ycf*1) to 9.565% (*pet*N–*psb*M). Again, ten of those regions showed considerably high values (*pi* > 5.7%; i.e., *rpl*32–*ndh*F, *trn*S–*trn*G, *ndh*E–*psa*C, *ndh*D–*ccs*A, *atp*F–*atp*H, *psb*M–*trn*D, *trn*E–*trn*T, *pet*L–*pet*G, *rps*16–*trn*Q, *acc*D–*psa*I; see Figure 4). A comparison of DNA sequence divergence revealed that three of these ten noncoding regions, *ndh*F–*rpl*32 (PICs = 96), *psb*M–*trn*D (PICs = 73) and *trn*S–*trn*G (PICs = 49), are the most variable regions across Stemonaceae (Figure A1). Thus, these three regions may be good candidates for resolving future low-level phylogeny and phylogeography in Stemonaceae. In a previous study, the availability of plastid noncoding regions was compared across 10 major lineages of angiosperms (such as Nymphaeales, monocots, eurosids) [44]. However, only five families of monocots represented by five species pairs were included, without Stemonaceae. The three variable regions predicted here are among the top 13 regions of monocots in the research by Shaw et al. [44], with *ndh*F–*rpl*32, *psb*M–*trn*D and *trn*S–*trn*G ranked first, third and 11th, respectively. Of these regions, *ndh*F–*rpl*32 has long been a popular region in phylogenetic studies of angiosperms [44,45]. Meanwhile, *psb*M–*trn*D and *trn*S–*trn*G are also noted as highly variable in Liliaceae [46] and occasionally used in low-level phylogenetic analyses (*Scabiosa*: [47]; *Solms-laubachia*: [48]). The resolution of recent divergences in monocots would benefit considerably by the inclusion of any or all of these highly variable regions.

### 2.4. Repetitive Sequences and SSR Polymorphisms

**Figure 5:**
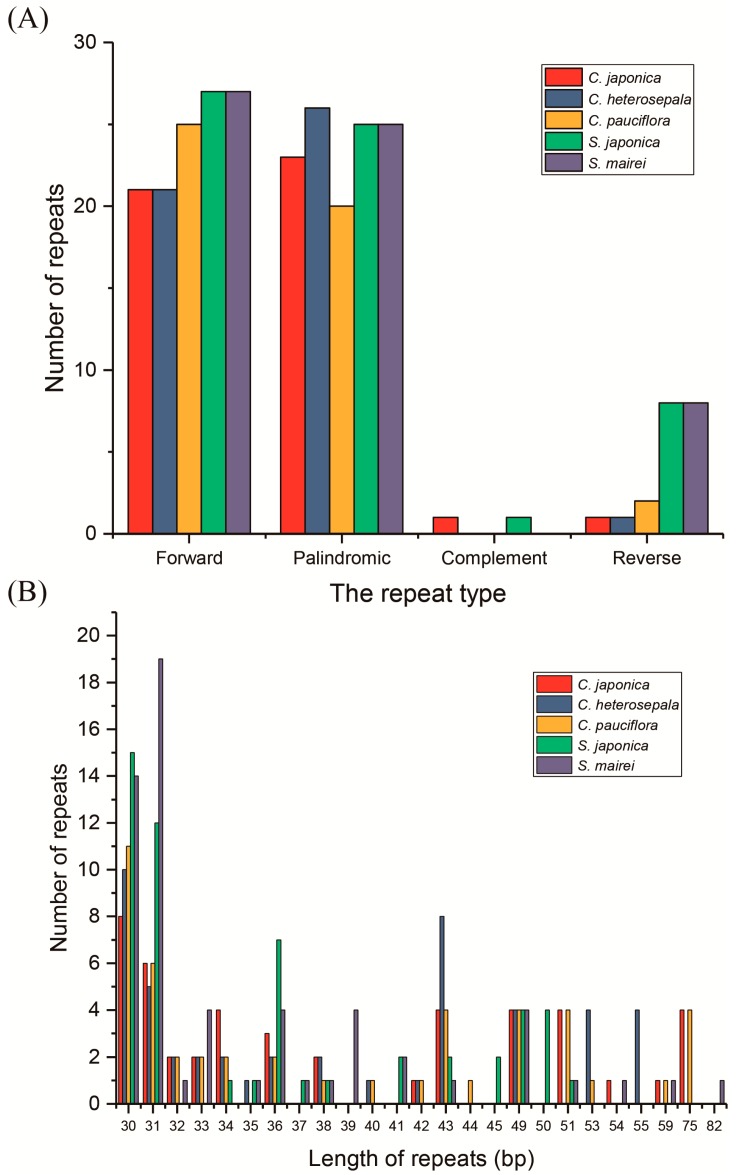
Analysis of repeated sequences in five Stemonaceae chloroplast genomes. (**A**) Frequency of repeats by length; (**B**) frequency of repeat types.

With the criterion of a copy size of 30 bp or longer and a sequence identity >90%, REPUTER [49] identified 47, 49, 48, 61 and 60 repeats (including forward, palindromic, complement and reverse repeats) in five cp genome sequences of *C. japonica*, *C. heterosepala*, *C. pauciflora*, *S. japonica* and *S. mairei*, respectively (Figure 5A). *C. japonica* contained 21 forward repeats, 24 palindromic repeats, 1 complement repeat and 1 reverse repeat, and *S. japonica* contained 27, 25, 1 and 8 repeats, correspondingly. The other two *Croomia* species and *S. mairei* contained no complement repeats. The numbers of forward repeats, palindromic and reverse repeats were, respectively, 21, 27 and 1 in *C. japonica*, 25, 21 and 2 in *C. heterosepala* and 27, 25 and 8 in *S. mairei* (Figure 5A). The lengths of majority repeats were 30, 31 and 43 bp in size (Figure 5B). For *Croomia*, the repeats were mainly located in *ycf*2 (46.8–58.3%) and non-coding regions (27.1–38.3%). As for *Stemona*, the repeats were mostly located in non-coding regions (58.3–59.0%) and *ycf*2 (33.3–34.4%). Only one repeat was across IGS (*psb*C–*trn*S) and CDS (*trn*S^UGA^). The remaining repeats were found located in genes such as *ccs*A, *ycf*1, *trn*G^UGA^, *trn*S^GGA^, *trn*S^GCU^ and *psa*B.

**Figure 6:**
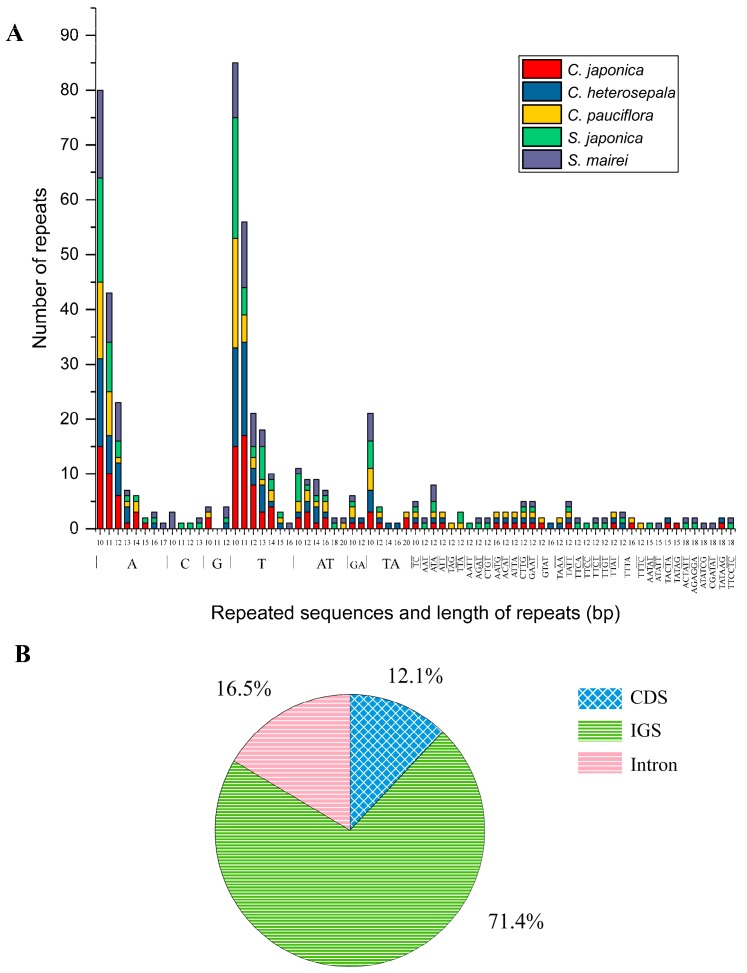
Simple Sequence Repeats (SSRs) in five Stemonaceae chloroplast genomes. (**A**) Numbers of SSRs by length; (**B**) distribution of SSR loci. IGS: intergenic spacer region; CDS: protein-coding regions.

SSRs in the cp genome present high diversity in copy numbers, and they are important molecular markers for plant population genomics and evolutionary history [50,51]. SSRs (≥10 bp) were detected in these five Stemonaceae cp genomes by MIcroSAtellite (MISA) analysis [52], ranging from 90–116 in total. Among these SSRs, the mononucleotide repeat unit (A/T) occupied the highest proportion, with 71.2% in *C. japonica*, 70.9% in *C. heterosepala*, 63.3% in *C. pauciflora*, 64.0% in *S. japonica* and 62.4% in *S. mairei* (Figure 6A). SSR loci were mainly located in IGS (71.4%) (Figure 6B) and were also detected in introns (16.5%) and CDS (12.1%), such as *mat*K, *atp*A, *rpo*C2, *rpo*B, *cem*A, *psb*F, *ycf*2, *ycf*1 and *ndh*D. In general, the SSRs of these five cp genomes showed great variation, which can be used in population genetic studies of *Croomia* and *Stemona* species.

### 2.5. Phylogenetic Analysis, Divergence Time and Ancestral Area Reconstruction

**Figure 7:**
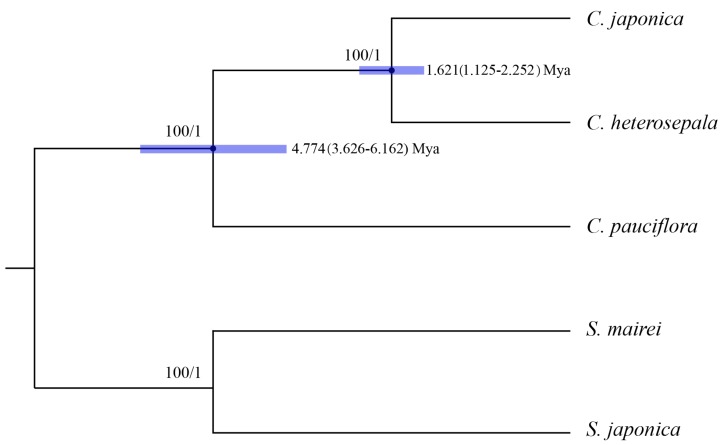
Phylogenetic relationships of three *Croomia* species inferred from Maximum Likelihood (ML) and Bayesian Inference (BI) and divergence time of three *Croomia* species estimated using Bayesian Evolutionary Analysis Sampling Trees (BEAST) analysis. Numbers above the lines represent ML bootstrap values and BI posterior probability. Blue bars indicate the 95% highest posterior density (HPD) credibility intervals for node ages (Mya). Numbers at the node represent divergence time (Mya) and 95% highest posterior density intervals. The phylogenetic tree based on 74 protein-coding genes is completely consistent with this topology.

CP genome sequences have been successfully used in angiosperm phylogenetic studies [22,53]. The Maximum Likelihood (ML) and Bayesian Inference (BI) analyses of both whole sequences and protein-coding region of three *Croomia* and two *Stemona* cp genomes yielded nearly identical tree topologies, with 100% bootstrap and 1.0 Bayesian posterior probabilities at each node (Figure 7). This phylogenetic tree supports the monophyly of *Croomia*. Two Asian species *C. japonica* and *C. heterosepala* formed a clade, being strongly recovered as sisters of the North American species *C. pauciflora*. This tree topology is largely congruent with that inferred from *trn*L–F [12], but obtained much higher bootstrap support values. Using average substitution rates of whole cp genomes, the divergence time between the two Asian species, *C. japonica* and *C. heterosepala*, was estimated as ca. 1.621 Mya (1.125–2.251 Mya) and, thus, compatible with the early-Pleistocene event. By contrast, the divergence time between North American *C. pauciflora* and Asian species was estimated as ca. 4.774 Mya (3.626–6.162 Mya) (i.e., the Late Miocene). The divergence times estimated in this paper are much older than that estimated by the strict molecular clock method (*C. pauciflora*/the East Asian lineage: ca. 2.61–0.41 Mya; *C. japonica*/*C. heterosepala*: ca. 0.84–0.13 Mya) [12].

**Figure A2:**
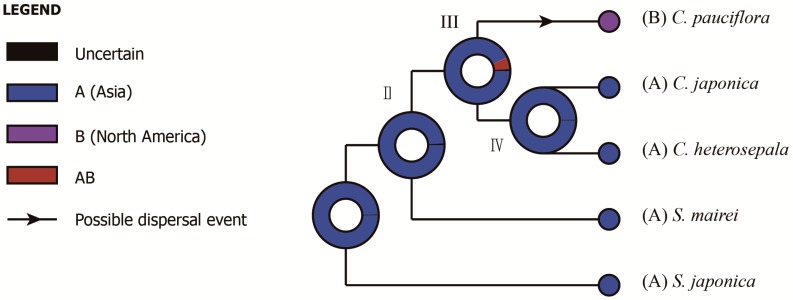
The Bayesian Binary MCMC (BBM) analysis of ancestral area reconstruction.

The divergence between *C. pauciflora* and two Asian species coincides with the first sundering of the Bering Land Bridge (BLB) between the late Miocene and early Pliocene, most approximately at 5.4–5.5 Mya (Milne and Abbott, 2002) [54]. The Bayesian Binary MCMC (BBM) analysis of ancestral area reconstruction identified Asia as the most likely ancestral range (Node III, marginal probability: 0.93; Figure A2), indicating a possible intercontinental plant migration from Asia to North America. Indeed, the BLB served as an important route for temperate floristic exchanges between Asia and North America from the Eocene to the early Pliocene [55,56]. Subsequently, as a member of the Tertiary relict flora [15], *Croomia* species on the two continents experienced disjunct distribution and evolved separately after the Late Miocene. Thus, we conclude that the current distribution and differentiation of *Croomia* species in eastern Asia and eastern North America likely resulted from a combination of ancient migration and vicariant events. The divergence time between *C. japonica* and *C. heterosepala* fell into the Early Pleistocene. Habitat fragmentation resulting from the climatic vicissitudes of the (Late) Quaternary likely led to the speciation of *C. japonica* and *C. heterosepala* [12]. The above inferences seem to be consistent with the palaeovegetational and climatic history of eastern Asia and eastern America. However, considering that the cp genome is a haploid, uniparentaly-inherited and single locus [57], a nuclear (biparental) marker is also needed to elucidate the diversification process and demography history of *Croomia* species.

## 3. Materials and Methods 

### 3.1. Sample Preparation, Sequencing, Assembly and Validation

Fresh leaves of *C. japonica* from China, *C. heterosepala* from Japan, *C. pauciflora* from North America and two outgroup species *Stemona japonica* (Bl.) Miq. and *S. mairei* (Levl.) Krause from China were sampled and dried with silica gel. The voucher specimens were deposited in the Herbarium of Zhejiang University (HZU). Total genomic DNA was extracted from ~3 mg materials using DNA Plantzol Reagent (Invitrogen, Carlsbad, CA, USA) following the manufacturer’s protocol. The quality and concentration of the DNA were detected using agarose gel electrophoresis. Purified DNA was sheared into ~500-bp fragments, and the fragmentation quality was checked on a Bioanalyzer 2100 (Agilent Technologies, Santa Clara, CA, USA). Paired-end sequencing libraries were constructed according to the Illumina standard protocol (Illumina, San Diego, CA, USA). Genomic DNAs of five species were sequenced using an Illumina HiSeq^TM^ 2000 (Illumina, San Diego, CA, USA) at Beijing Genomics Institute (BGI; Shenzhen, China). Plastome sequences were assembled using a combination of de novo and reference-guided assembly [58]. Firstly, to obtain clean reads, the CLC-quality trim tool was used to remove low-quality bases (*Q* < 20, 0.01 probability error). Secondly, we assembled the clean reads into contigs on the CLC de novo assembler. Thirdly, all the contigs were aligned with the reference cp genome of *Carludovica palmate* Ruiz. & Pav. (NC_026786.1) using local BLAST (http://blast.ncbi.nlm.nih.gov/) (27 December 2016), and aligned contigs were ordered according to the reference cp genome with ≥90% similarity and query coverage. Then, to construct the draft cp genome of each species, the ordered contigs usually representing the whole reconstructed genome were imported into GENEIOUS v9.0.5 software (http://www.geneious.com) (18 March 2017), where the clean reads were remapped onto the contigs.

### 3.2. Genome Annotation and Whole Genome Comparison

The annotation of five species was performed using the Dual Organellar GenoMe Annotator (DOGMA) [59]. The start and stop codons and intron/exon boundaries were manually corrected by comparison to homologous genes from the reference genome of *C. palmate*. We also verified the transfer RNAs (tRNAs) using tRNAscan-SE v1.21 with default settings [60]. The circular genome maps were drawn using the OrganellarGenome DRAW tool (OGDRAW) [61], followed by manual modification.

Genome comparison among the five Stemonaceae cp genomes was analyzed using mVISTA [62] with *C. palmate* as a reference. Six genome sequences were aligned in Shuffle-LAGAN mode with default parameters, and the conservation region was visualized in an mVISTA plot. To identify the divergence hotspot regions in the five Stemonaceae cp genomes, the nucleotide variability of protein coding genes, introns and intergenic spacer sequences of five species were evaluated using DNASP v5.10 [63]. The above regions were extracted following two criteria: (a) total number of mutation (Eta) >0; and (b) the aligned length >200 bp. The inverted regions in *cem*A, *cem*A–*pet*A and *pet*A were excluded. The top ten most variable noncoding regions with a high Pi value were counted by Potentially Informative Characters (PICs) across species pair of *C. japonica* and *S. japonica* following Shaw et al. [64]. Any large structural event of the cp genome, such as gene order rearrangements or IR expansion/contractions, were recorded. 

### 3.3. Characterization of Repeat Sequence and SSRs

REPUTER [49] was used to find the location and length of repeat sequences, including forward, palindrome, complement and reverse repeats in the five cp genomes. The minimum repeat size was set to 30 bp, and the sequence identity of repeats was no less than 90% or greater sequence identity with the Hamming distance equal to 3. The MISA perl script was used to detect simple sequence repeats (SSRs) [52] with thresholds of 10 bp in length for mono-, di-, tri, tetra-, penta- and hexa-nucleotide SSRs.

### 3.4. Phylogenetic Analysis, Divergence Time and Ancestral Area Reconstruction

The five cp genome sequences were aligned using MAFFT v7 [65]. Two *Stemona* species were used as outgroups. ML and BI analysis were used to reconstruct the phylogenetic trees. In order to examine the phylogenetic utility of different regions, two datasets were used: (1) the complete cp genome sequences; (2) 78 protein-coding genes shared by the five cp genomes (two inverted genes of *cem*A and *pet*A in *Stemona* species were excluded). Gaps (indels) were treated as missing data. The Akaike Information Criterion (AIC) in JMODELTEST v2.1.4 [66] was used to determine the best-fitting model of nucleotide substitutions. The GTR + I + G model was used for two datasets. The ML tree was constructed using RAXML-HPC v8.2.10 with 1000 replicates on the Cyberinfrastructure for Phylogenetic Research (CIPRES) Science Gateway website (http://www.phylo.org/) (10 May 2017) [67]. BI analysis was conducted in MRBAYES v3.2 [68]. The Markov chain Monte Carlo (MCMC) was set to run 1,000,000 generations and sampled every 1000 generations. The first 25% of generations was discarded as burn-in. 

Due to the lack of fossil records, we used the average substitution rate 0.51952 × 10^−9^ per site per year (s/s/y) of the whole cp genome in Brassicaceae [69,70] to estimate interspecific divergence time of *Croomia*. The Bayesian analysis was implemented in BEAST v1.8.4 [71] using the GTR + I + G substitution model. MCMC analysis of 20,000,000 generations was implemented, in which every 1000 generations were sampled, under an uncorrelated lognormal relaxed clock approach using the Yule speciation tree prior with the substitution rate. TRACER v1.6 [72] was used to check the effective population size (ESS) >200. TREEANNOTATOR v.1.8.4 [73] was used to produce maximum clade credibility trees from the trees after burning-in of 25%. The final tree was visualized in FIGTREE v1.4.3 (http://tree.bio.ed.ac.uk/software/figtree/) (13 May 2017). 

To reconstruct the historical biogeography of *Croomia*, we performed Bayesian Binary MCMC (BBM) analysis as implemented in RASP v3.1 [74] using trees retained from the BI analysis (see above). According to the distribution of *Croomia*, we defined the following two areas: A, Asia (East Asia/South Asia); and B, North America. Accounting for phylogenetic uncertainty, we used 500 trees randomly chosen across all post-burn-in trees generated from BEAST analysis and ran the BBM analysis. A fixed JC + G (Jukes–Cantor + Gamma) model was chosen with a null root distribution. The MCMC chains were run for 500,000 generations, and every 100 generations were sampled. The ancestral ranges obtained were projected onto the MCC tree. 

## 4. Conclusions

Here, we sequenced the first five complete cp genomes in Stemonaceae. Each genome possesses the typical structure shared with other angiosperm species. Several highly variable noncoding cpDNA regions were identified, which should be the best choices for future phylogenetic, phylogeographic and population-level genetic studies in Stemonaceae. The phylogenomic and biogeographic analyses of *Croomia* reveal that ancient migration and vicariance-driven allopatric speciation resulting from historical climate oscillations most likely played roles in the formation of the disjunct distributions and divergence of these three *Croomia* species.

## Figures and Tables

**Table 1:** List of genes in Stemonaceae chloroplast genomes.

Category of Genes	Groups of Genes	Names of Genes
Self-replication	rRNA genes	*rrn16*(×2), *rrn23*(×2), *rrn4.5*(×2), *rrn 5*(×2)
	tRNA genes	*trnA-UGC* *(×2), *trnC-GCA*, *trnD-GUC*, *trnE-UUC*, *trnF-GAA*, *trnfM-CAU*, *trnG-GCC*, *trnG-UCC* *, *trnH-GUG*(×2), *trnI-CAU*(×2), *trnI-GAU* *(×2), *trnK-UUU* *, *trnL-CAA*(×2), *trnL-UAA* *, *trnL-UAG*, *trnM-CAU*, *trnN-GUU*(×2), *trnP-UGG*, *trnQ-UUG*, *trnR-ACG*(×2), *trnR-UCU*, *trnS-GCU*, *trnS-GGA*, *trnS-UGA*, *trnT-GGU*, *trnT-UGU*, *trnV-GAC*(×2), *trnV-UAC* *, *trnW-CCA*, *trnY-FUA*
	Small subunit of ribosome	*rps2*, *rps3*, *rps4*, *rps7*(×2), *rps8*, *rps11*, *rps12* **(×2), *rps14*, *rps15*, *rps16* *, *rps18*, *rps19*(×2)
	Large subunit of ribosome	*rpl2* *(×2), *rpl14*, *rpl16* *, *rpl20*, *rpl22*(×2), *rpl23*(×2), *rpl32*, *rpl33*, *rpl36*
	DNA-dependent RNA polymerase	*rpoA*, *rpoB*, *rpoC1* *, *rpoC2*
Genes for photosynthesis	Subunit of NADH-dehydrogenase	*ndhA* *, *ndhB* *(×2), *ndhC*, *ndhD*, *ndhE*, *ndhF*, *ndhG*, *ndhI*, *ndhH*, *ndhJ*, *hdhK*
	Subunit of Photosystem 1	*psaA*, *psaB*, *psaC*, *psaI*, *psaJ*, *ycf3* **
	Subunit of Photosystem 2	*psbA*, *psbB*, *psbC*, *psbD*, *psbE*, *psbF*, *psbH*, *psbI*, *psbJ*, *psbK*, *psbL*, *psbM*, *psbN*, *psbT*
	Subunits of cytochrome b/f complex	*petA*, *petB* *, *petD* *, *petG*, *petL*, *petN*
	Subunits of ATP synthase	*atpA*, *atpB*, *atpE*, *atpF* *, *atpH*, *atpI*
	Large subunit of rubisco	*rbcL*
Other genes	Maturase	*matK*,
	Protease	*clpP* **
	Envelope membrane protein	*cemA*
	Subunit of Acetyl-CoA-carboxylase	*accD*
	c-type cytochrome synthesis gene	*ccsA*
	Translation initiation factor IF-1	*infA*
Genes of unknown function	Open reading frames (ORF, ycf)	*ycf1*, *ycf2*(×2), *ycf4*, *lhbA*

* Gene with one intron, ** gene with two introns; (×2) indicates genes duplicated in the IR region.

## Supplementary Materials

Supplementary materials can be found at http://www.mdpi.com/1422-0067/19/8/2383/s1.

## Abbreviations

cp	Complete chloroplast
IR	Inverted Repeat
lSC	Large Single Copy
SSC	Small Single Copy
Pi	Nucleotide variability
SSR	Simple Sequence Repeat
PIC	Potentially Informative Character
ML	Maximum likelihood
BI	Bayesian Inference
MCMC	Markov chain Monte Carlo
