# Supplementary material for: Phylogenomic and Comparative Analyses of Complete Plastomes of Croomia and Stemona (Stemonaceae)
Source: Int J Mol Sci. 2018 Aug 13;19(8):2383. doi: 10.3390/ijms19082383 (PMC6122011; doi:10.3390/ijms19082383)
Supplement: Supplementary file 1 [file ijms-19-02383-s001.pdf]

Table S1. The basic characteristics of five Stemonaceae chloroplast genomes.

Characteristics	*C. japonica*	*C. heterosepala*	*C. pauciflora*	*S. japonica*	*S. mairei*
Clean reads	29,120,480	14,478,324	31,094,272	17,784,508	14,163,520
Average reads length (bp)	125	150	125	150	150
Number of contigs	83,092	50,369	111,071	105,805	123,479
Total length of contigs	29,978,872	19,017,346	47,503,502	41,588,737	45,904,433
Total cpDNA size (bp)	154,672	154,407	155,261	154,224	154,307
LSC length (bp)	81,981	81,844	82,429	82,117	82,254
SSC length (bp)	18,271	18,145	18,346	17,943	17,889
IR length (bp)	27,210	27,210	27,243	27,082	27,082
Total CDS length (bp)	79,452	79,488	79,611	79,398	79,239
Total tRNA length (bp)	2877	2877	2877	2877	2901
Total rRNA length (bp)	9052	9052	9052	9060	9060
Total GC content (%)	38.3	38.3	38.3	38.0	38.0
LSC	36.6	36.6	36.6	36.2	36.2
SSC	32.3	32.3	32.5	32.1	32.1
IR	42.8	42.8	42.9	42.7	42.7
Total number of genes	134	134	134	134	134
Protein-coding genes	80	80	80	80	80
tRNA genes	30	30	30	30	30
rRNA genes	4	4	4	4	4
Duplicated genes	20	20	20	20	20

Table S2. The location information of the five Stemonaceae species.

**Species**	**Location**	**Latitude**	**Longitude**
Croomia japonica	Hangzhou, China	30.3303	119.4377
Croomia heterosepala	Nara, Japan	34.5166	135.6833
Croomia pauciflora	Alabama, USA	32.8605	−85.7975
Stemona japonica	Hangzhou, China	30.2283	119.1544
Stemona mairei	Zhongdian, China	27.8231	99.7060
